# Diaphragmatic endometriosis with hepatic herniation: case report and review of literature

**DOI:** 10.3389/fmed.2026.1740350

**Published:** 2026-04-01

**Authors:** Katarzyna Pietrzak, Anna Weronika Szablewska, Aleksandra Gaworska-Krzemińska, Bartosz Pryba

**Affiliations:** 1 Independent Monoprofile Medical Simulation Laboratory, Institute of Nursing and Midwifery, Faculty of Health Sciences with the Institute of Maritime and Tropical Medicine, Medical University of Gdańsk, Gdańsk, Poland; 2 Department of Obstetric and Gynecological Nursing, Institute of Nursing and Midwifery, Faculty of Health Sciences with the Institute of Maritime and Tropical Medicine, Medical University of Gdańsk, Gdańsk, Poland; 3 Division on Nursing Management, Institute of Nursing and Midwifery, Faculty of Health Sciences with the Institute of Maritime and Tropical Medicine, Medical University of Gdańsk, Gdańsk, Poland

**Keywords:** case report, catamenial pneumothorax, diaphragmatic hernia, thoracic endometriosis, video-assisted thoracic surgery

## Abstract

Diaphragmatic endometriosis is a rare and often underrecognized manifestation of extrapelvic endometriosis that may lead to severe complications such as diaphragmatic rupture or herniation. We report the case of a 42-year-old woman with a long history of cyclical thoracic pain who was finally diagnosed with right-sided diaphragmatic endometriosis, complicated by a large diaphragmatic defect and hepatic herniation. For more than two decades, her symptoms were misattributed to musculoskeletal causes, resulting in delayed diagnosis. Imaging revealed a right diaphragmatic defect with protrusion of the liver into the thoracic cavity, and video-assisted thoracoscopic surgery (VATS) confirmed the presence of ectopic endometrial glands as well as stroma positive for CD10 and estrogen/progesterone receptors. Surgical repair with diaphragmatic reconstruction was performed. However, the postoperative course was complicated by catamenial pneumothorax, requiring a second VATS procedure with pleurectomy and pleural abrasion Subsequent hormonal therapy resulted in partial symptom control. This case highlights the diagnostic complexity and progressive nature of diaphragmatic endometriosis and underscores the importance of early multidisciplinary collaboration between gynecologic and thoracic specialists. It further demonstrates that surgical repair alone may be insufficient in cases with thoracic involvement and that coordinated multidisciplinary management is essential to improve long-term disease control.

## 1 Background

Endometriosis, defined as the presence of endometrial-like tissue outside the uterine cavity, affects approximately 6–10% of women at a reproductive age globally, with higher prevalence in those presenting with chronic pelvic pain or infertility. While the pelvis remains the most common site for this disorder, extrapelvic manifestations occur in up to 12% of women with endometriosis, with the thoracic cavity representing thoracic endometriosis the most frequent non-abdominopelvic location (1, 2). Thoracic Endometriosis Syndrome (TES) refers to a set of clinical manifestations suggesting intrathoracic endometriosis—such as catamenial pneumothorax, hemothorax, hemoptysis, chest pain or pulmonary nodules-that are temporally associated with menstruation but without histopathological confirmation. In contrast, a definitive diagnosis of thoracic endometriosis (TE) requires histological or immunohistochemical evidence of endometrial tissue within the thoracic cavity, such as through biopsy obtained during video-assisted thoracoscopic surgery (3–5).

The diagnostic work-up for suspected thoracic endometriosis begins with a detailed clinical history emphasizing the temporal association of symptoms with menstruation, supported by imaging such as chest X-ray, computed tomography (CT) or magnetic resonance imaging (MRI). Video-assisted thoracoscopic surgery (VATS) is both the gold standard for direct visualization and biopsy, enabling histopathological confirmation required for the diagnosis of TES (6–8).

In one large series, endometriotic lesions in thoracic endometriosis were most frequently found on the diaphragm (78.82%), on the pleura (14.33%) and the lungs (4.46%) (9, 10). Clinically, the hallmark presentations of thoracic endometriosis as well as thoracic endometriosis syndrome include catamenial pneumothorax (72%), hemothorax (13%), hemoptysis (10%) and pulmonary nodules (4%). Patients most frequently report symptoms such as chest or shoulder pain, dyspnea and cough, which characteristically coincide with the menstrual cycle (3).

Among these thoracic locations, diaphragmatic involvement is common but often overlooked. When symptoms do occur, they are often related to phrenic nerve irritation, causing referred pain to the periscapular area or neck, typically on the right side. In isolated cases, the condition may manifest as cyclical pain in the neck, shoulder, right upper quadrant or epigastric region. Repeated cyclical necrosis of the diaphragmatic tissue is thought to result in small defects, which may gradually enlarge and merge, potentially leading to hernia formation. In rare instances, diaphragmatic rupture may be the initial presentation of thoracic endometriosis. The prevalence of diaphragmatic endometriosis ranges from 0.67 to 4.7%, and while most lesions are superficial, full-thickness involvement and consequent diaphragmatic defects may lead to the herniation of abdominal contents into the thoracic cavity (11–13).

Effective multidisciplinary collaboration plays an essential role in both the recognition and management of Thoracic Endometriosis Syndrome (TES) and its rare complications. The coordinated efforts of specialists in gynecology, pulmonology, thoracic surgery, radiology, physiotherapy and psychology are critical for identifying cyclical, multisystem symptoms, ensuring timely diagnosis and optimizing patient outcomes. In this case report, the importance is underscored of a patient-centered, interdisciplinary approach in managing a rare and complex presentation-thoracic endometriosis complicated by a diaphragmatic hernia, and highlighted is the value of close communication and integrated decision-making across specialties in achieving comprehensive care. This work aims to address a notable gap in the literature regarding diaphragmatic endometriosis leading to herniation—a rare and underrecognized manifestation. By combining a detailed case description with a concise literature review, we emphasize the potential mechanisms, diagnostic pitfalls and multidisciplinary management strategies relevant to such presentations. This dual approach not only adds to the limited clinical evidence but also provides practical implications for surgical planning and long-term care in patients with advanced extrapelvic endometriosis (14–16).

## 2 Case presentation

This case report was prepared in accordance with CARE guidelines to ensure comprehensive and transparent reporting of clinical information. A 42-year-old woman, mother of two children delivered vaginally, with a history of one miscarriage, and longstanding cyclical thoracic pain was diagnosed with right-sided diaphragmatic endometriosis complicated by a diaphragmatic hernia, following years of delayed recognition. She had no chronic comorbidities; her family history was notable for maternal hypertension and paternal diabetes mellitus. Her symptoms, which included chest pain, cough and a sensation of shifting air during menstruation, were initially misattributed to musculoskeletal issues. The diagnostic process of endometriosis in this patient occurred in several stages. Cyclical interscapular pain began in 2000, but no underlying cause was identified for more than a decade. In 2012, surgical excision of a small umbilical lesion led to a histopathological diagnosis of umbilical endometriosis. At that time, the gynecological evaluation, including transvaginal ultrasound, did not expose any additional lesions, and the condition was initially considered limited to the umbilicus. Six years later, in 2018, a routine pelvic ultrasound performed by the same gynecologist revealed an irregular mass suspicious for malignancy. This finding prompted referral to a specialist experienced in endometriosis management. Further assessment, with dedicated pelvic magnetic resonance imaging (MRI) and expert transvaginal ultrasonography, demonstrated extensive deep infiltrating endometriosis, affecting multiple pelvic organs. These discoveries confirmed the systemic nature of the disease and provided the basis for subsequent multidisciplinary management.

### 2.1 Assessment via physical examination

On presentation, the patient was hemodynamically stable with normal vital signs. Chest inspection revealed a mild pectus excavatum deformity, without evidence of respiratory distress. Palpation elicited mild tenderness over the right lower anterior chest wall and costal margin, which intensified during menstruation. Percussion of the right lower thoracic area was slightly dull, and breath sounds were diminished at the right lung base. During symptomatic episodes, she reported cyclical right-sided chest and shoulder pain radiating to the scapular area, occasionally associated with mild dyspnea and dry cough. Abdominal examination revealed mild right upper quadrant tenderness, without palpable organomegaly or herniation. Prior to definitive surgical management, symptom severity was retrospectively quantified using a 10-point visual analog scale (VAS). The patient reported severe dysmenorrhea before hormonal suppression (VAS 7–8/10), dyspareunia (VAS 7/10), and chronic pelvic pain (VAS 4/10). She denied dyschezia and dysuria, except for transient burning during urinary tract infections. Thoracic pain was cyclical and progressively intensified over the years, ranging from 5 to 6/10 in earlier stages to 10/10 during severe preoperative episodes requiring analgesics.

### 2.2 Imaging findings

**Figure 1:**
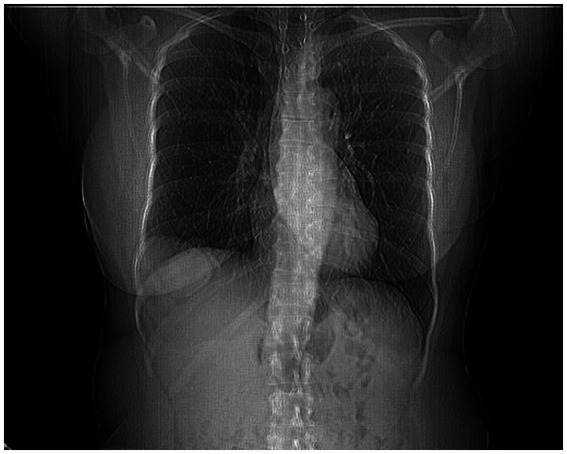
Coronal CT image showing right diaphragmatic hernia with herniation of the liver into the thoracic cavity.

Computed tomography (CT) of the chest and upper abdomen revealed an elevated and irregular right hemidiaphragm (Figure 1), with a focal protrusion of hepatic parenchyma (segments VII and VIII) into the thoracic cavity, measuring approximately 68 × 38 mm. A 10 × 18 mm hypodense lesion was noted at the periphery of hepatic segment VIII, of indeterminate character on a single-phase scan. No pulmonary consolidations or suspect nodules were present. These findings raised the suspicion of a diaphragmatic hernia-either congenital or post-traumatic in origin and further evaluation by a thoracic surgeon as well as magnetic resonance imaging (MRI) was recommended.

**Figure 2:**
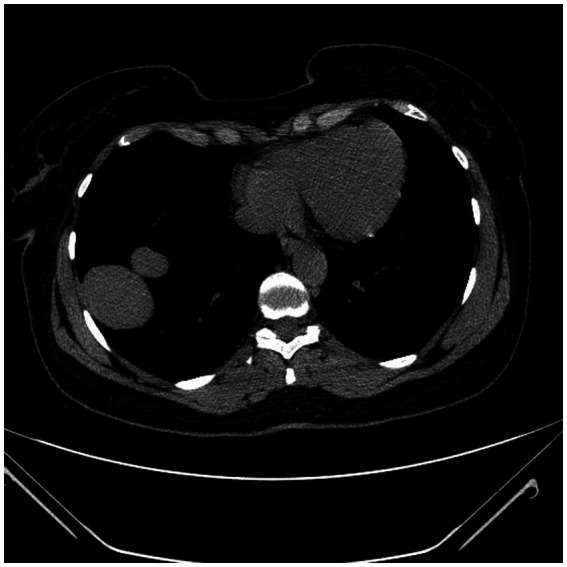
Axial CT image showing right diaphragmatic defect with herniation of the liver into the thoracic cavity.

**Figure 3:**
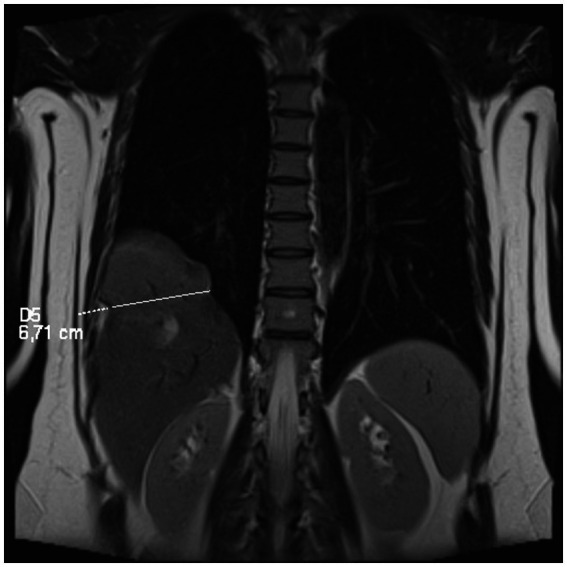
MRI image of liver herniation.

MRI confirmed a posterior diaphragmatic defect with hepatic herniation but did not demonstrate anatomical features typical of congenital diaphragmatic hernias. An elevated right hemidiaphragm and a posterior diaphragmatic defect was confirmed through which a portion of the right hepatic lobe (48 × 74 × 76 mm) herniated into the thoracic cavity (Figure 2). The margins of the diaphragm at the defect site were poorly visualized, with a herniation width of approximately 52 mm in the sagittal plane and 70 mm in the coronal one. The herniated hepatic tissue demonstrated a slightly increased T2 signal, suggesting edema. A trace amount of pleural fluid was present near the herniation site. Additionally, a 10 × 18 mm lesion in segment VIII showed gradual post-contrast enhancement, consistent with a cavernous hemangioma. Follow-up abdominal ultrasound showed interval enlargement of a hyperechogenic lesion in the right hepatic lobe, prompting further evaluation with contrast-enhanced MRI, which had been performed with the official report pending at the time of manuscript revision. No mediastinal or hilar lymphadenopathy was identified. The imaging findings were interpreted as suggestive of a right-sided diaphragmatic hernia, prompting referral to thoracic surgery for further evaluation and management. Limited access to advanced imaging and specialist consultations contributed to delayed diagnosis and prolonged symptom burden. Financial constraints and fragmented care pathways further hindered timely assessment and comprehensive evaluation of both pelvic and thoracic disease (Figure 3).

### 2.3 Therapeutic interventions

During thoracic surgical consultation, the patient reported a long-standing history of cyclical thoracic and shoulder pain temporally associated with menstruation, raising strong clinical suspicion of thoracic endometriosis syndrome. After more than two decades of progressively worsening symptoms, the diagnosis was confirmed via video-assisted thoracoscopic surgery (VATS). The procedure was performed using two port incisions and an additional utility incision protected by a wound retractor. Intraoperatively, a 6 × 8 cm diaphragmatic defect was identified, with partial herniation of the right hepatic lobe through the defect. The margins of the diaphragmatic defect were irregular and fibrotic rather than smooth or well circumscribed. In addition, multiple smaller defects were observed within the fibrous portion of the diaphragm that did not extend through its full thickness, suggesting chronic tissue weakening rather than an acute perforation. The herniated portion of the liver was covered by fibrotic tissue. Multiple small defects were noted within the fibrous portion of the diaphragm that did not extend through its full thickness. A bluish-black nodule—measuring approximately 1 cm—was observed on the diaphragm, and another of similar size within the pulmonary ligament. Scattered, firm, dark nodular lesions measuring <5 mm were identified over all pulmonary lobes. A subpleural nodule of about 1 cm was visualized in the lower lobe, and a small emphysematous bulla—a few millimeters in diameter—was present in the middle lobe. Two adjacent dark-gray nodules were detected on the parietal pleura near the third to fourth intercostal spaces. The hernia sac was carefully dissected, and diaphragmatic reconstruction was performed using four interrupted 5–0 Ticron sutures, followed by oversewing of the suture line with 2–0 Ticron for reinforcement. The diaphragmatic nodule was excised, and the site was reinforced with an additional 2–0 Ticron suture. The nodule within the pulmonary ligament and both parietal pleural nodules were also excised. Partial pleurectomy of the parietal pleura was carried out and complemented by pleural abrasion to promote adhesion. A subpleural nodule in the lower lobe was resected using a green Endo GIA stapler. Hemostasis was achieved, the pleural cavity was irrigated with povidone-iodine solution and a 28F chest drain was placed. The incisions were closed in layers and dressed in the standard manner.

**Figure 4:**
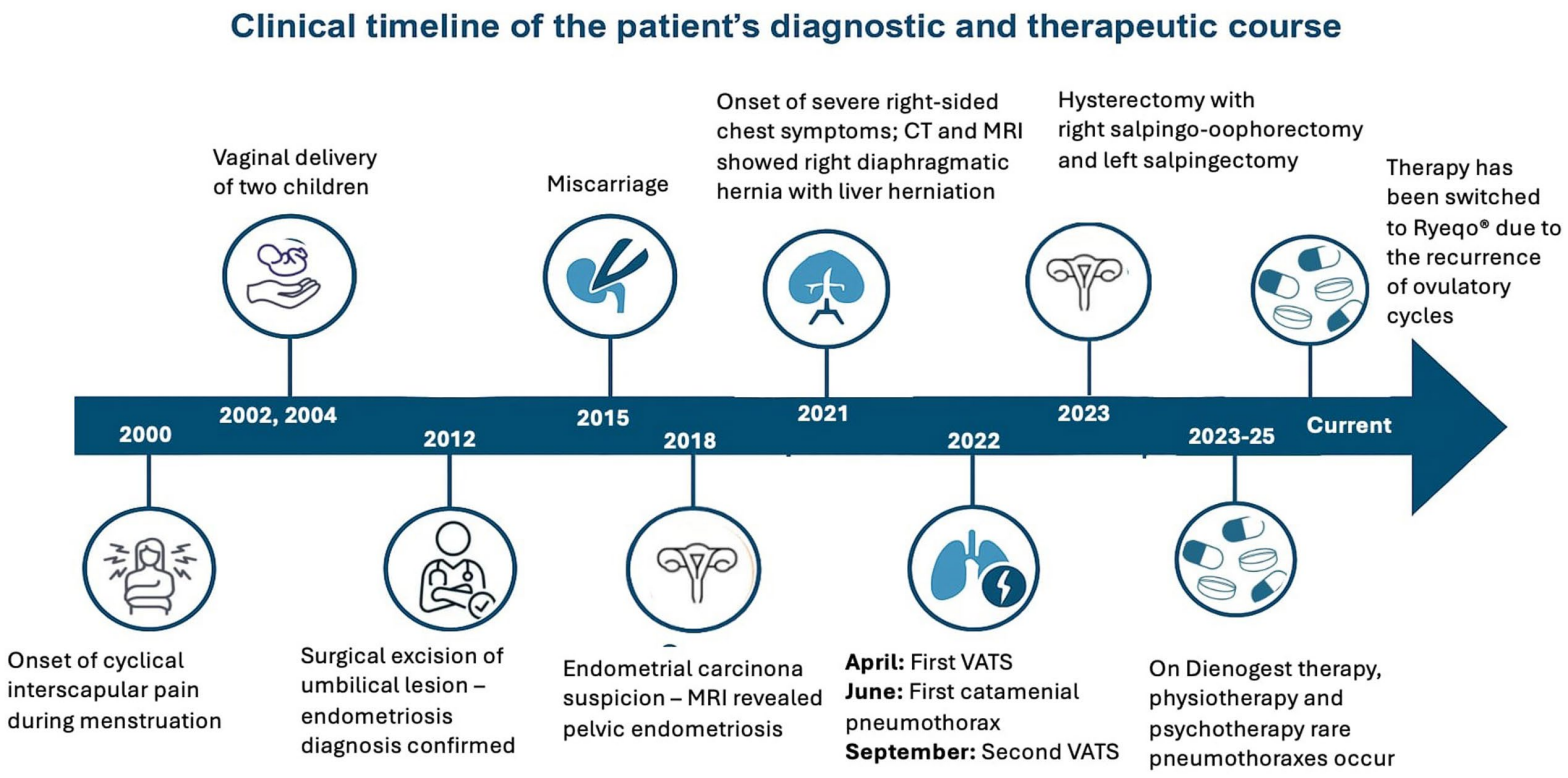
Patient’s medical history timeline.

Histopathological analysis of the diaphragmatic and pleural specimens obtained during video-assisted thoracoscopic surgery revealed foci of ectopic endometrial glands and stroma within the fibrotic diaphragmatic tissue. The endometrial stroma showed positive immunohistochemical staining for CD10, while the glandular epithelium was positive for estrogen and progesterone receptors (ER/PR), confirming the diagnosis of thoracic endometriosis (Figure 4).

The patient experienced her first episode of catamenial pneumothorax only after the diaphragmatic repair, having had no such events prior to surgery. Subsequently, she underwent a second VATS due to recurrent pneumothorax. A repeat video-assisted thoracoscopic surgery (VATS) was performed through a utility incision with a wound protector. The lung was found to be partially adherent to the parietal pleura. In the upper lobe, a thin-walled emphysematous bulla—measuring approximately 4 mm in diameter—was identified and resected using a purple Endo GIA stapler.

A small wedge resection of the upper lobe was also performed with the same device. Pleurectomy and mechanical pleural abrasion were carried out in all areas without adhesions to promote pleurodesis. Dense adhesions between the lung and diaphragm were preserved. Meticulous hemostasis was achieved, and an underwater air-leak test confirmed airtight staple lines. Through a utility port, 28F and 20F chest drains were inserted, and an intercostal catheter for postoperative analgesia was placed. The incisions were closed in layers and sterile dressings were applied.

Due to persistent pelvic pain and imaging-confirmed deep infiltrating endometriosis, the patient was qualified for elective gynecologic laparoscopic surgery in September 2023. Intraoperatively, the uterus was enlarged and affected by diffuse adenomyosis. Both ovaries were adherent posteriorly in a “kissing ovaries” configuration with obliteration of the pouch of Douglas. The right ovary appeared cystically altered, while the left ovary was macroscopically preserved. Deep infiltrating endometriosis involving the left uterosacral ligament was identified and excised. A total laparoscopic hysterectomy with right salpingo-oophorectomy and left salpingectomy was performed. No macroscopic bowel or rectovaginal septum infiltration was observed intraoperatively.

Based on intraoperative findings complemented by preoperative MRI assessment, the extent of pelvic disease was classified according to the #Enzian 2021 system as: #Enzian(s) O0/1, T3/3, B2/0, F(diaphragm), F(umbilicus) (17).

The T3 score reflects bilateral tubo-ovarian adhesions with a “kissing ovaries” configuration and obliteration of the pouch of Douglas. The B2 score corresponds to a 1–3 cm deep infiltrating lesion of the left uterosacral ligament. The classification was primarily based on surgical findings (s). The absence of deep infiltrating endometriosis in compartments A (rectovaginal septum) and C (bowel) was confirmed by preoperative magnetic resonance imaging, as these structures were not described as involved in the operative report (17).

Hormonal therapies and hysterectomy have helped stabilize the patient’s symptoms. Currently, she continues to have small pneumothoraxes between pleural adhesions, which—although still uncomfortable—occur only about once every 6 months and no longer significantly limit daily functioning. The main drawback of these episodes is their slow resolution time, which tends to shorten when she reduces workload and physical activity, focusing mainly on breathing exercises and a generally energy-conserving lifestyle—an approach she has gradually learned to adopt.

### 2.4 Pharmacological treatment

The patient has been on continuous hormonal therapy since the age of 18 to manage dysmenorrhea and endometriosis-related pain. Initial combined oral contraceptives (1998–2001) provided symptom relief and were discontinued for pregnancies (2001–2006). Subsequent use of desogestrel-only pills (Cerazette) and the transdermal patch (Evra^®^) offered partial benefits but caused irregular bleeding and local irritation, leading to insertion of a levonorgestrel intrauterine system (LNG-IUS, Mirena^®^) in 2009. The first and second LNG-IUS (2009–2014; 2016–2021) both achieved good pain and bleeding control. After a recurrence of heavy bleeding, a third Mirena^®^ was inserted in early 2022. From January 2023 to September 2025, the patient additionally used a combined oral contraceptive (Diemono^®^) alongside the IUS, including perioperatively in 2023. Since September 2025, the therapy has been switched to Ryeqo^®^ (relugolix/estradiol/norethindrone) due to the recurrence of ovulatory cycles in the remaining ovary and the onset of a chest “grating” sensation, suggestive of possible thoracic endometriosis activity. The new regimen has resulted in satisfactory symptom control and improved quality of life, without relevant adverse effects. The complete chronology of the patient’s hormonal and pharmacological therapy—including treatment modifications over time—is available in the Supplementary materials.

### 2.5 Patient’s perspective

The patient describes her journey as a long and frustrating process of self-diagnosis, driven by lack of recognition and continuity in medical care. Although initial symptoms began in early adulthood, she received no indication that her recurring thoracic symptoms could be related to endometriosis. The majority of her insights came from personal research, often via online resources and international forums. She emphasizes the emotional and physical toll of not being believed, the difficulty of navigating healthcare systems and the sense of relief upon finally obtaining a definitive diagnosis. The condition has significantly impacted her professional life—particularly by not being able to travel by plane, which was previously an integral part of her work. Nonetheless, she reports feeling empowered by taking control of her health, supported by a multidisciplinary team and psychotherapy. She specifically notes the role of nursing staff in providing clear explanations of diagnostic and treatment procedures, monitoring for early signs of complications and offering emotional support during hospital stays. Education provided by nurses on symptom tracking, lifestyle adjustments and respiratory exercises contributed to her confidence in self-management after discharge. She also highlights the role of dietary and lifestyle modifications, hormonal therapy and physiotherapy in regaining stability. With ongoing support and monitoring, she has adapted to a new version of normality, but stresses that earlier recognition could have prevented years of suffering and complications.

This case illustrates the complex, delayed diagnostic course and highlights the need for greater awareness of thoracic endometriosis and its rare complications.

## 3 Discussion

The literature review was conducted as a narrative, targeted review to contextualize the presented case within previously reported cases of diaphragmatic and thoracic endometriosis. Relevant publications were identified through searches of PubMed, Scopus, Embase, and Web of Science. The search strategy included combinations of keywords such as “diaphragmatic endometriosis,” “thoracic endometriosis,” “diaphragmatic hernia,” “hepatic herniation” and related terms. Priority was given to case reports, case series, and review articles published in English that addressed clinical presentation, diagnostic challenges, and surgical management. Given the rarity of the condition, no formal inclusion or exclusion criteria were applied.

### 3.1 Clinical and diagnostic considerations

This case underscores an uncommon manifestation of thoracic endometriosis complicated by a diaphragmatic hernia, emphasizing the diagnostic complexity of extrapelvic endometriosis. Thoracic endometriosis, although the most frequent extrapelvic form remains underrecognized due to nonspecific symptoms and overlapping presentations with other thoracic disorders. The spectrum known as Thoracic Endometriosis Syndrome (TES) encompasses catamenial pneumothorax, hemothorax, hemoptysis and pulmonary nodules. Diaphragmatic involvement—as in this case—is often asymptomatic until advanced complications such as herniation occur. Our patient presented with a large right-sided diaphragmatic defect and hepatic herniation, initially mistaken for a congenital or post-traumatic hernia, illustrating how delayed recognition contributes to disease progression.

Although congenital or traumatic diaphragmatic hernia was initially considered based on computed tomography findings, the sequential diagnostic process argued against these etiologies. MRI did not reveal features typical of congenital defects, and the absence of a trauma history further reduced the likelihood of an acquired traumatic lesion. In contrast, the patient’s long-standing cyclical symptoms, intraoperative findings and histopathological confirmation supported an endometriosis-related mechanism.

### 3.2 Comparison with previously reported cases

In several reports, it has been supported that diaphragmatic endometriosis can evolve into herniation following incomplete or absent repair Islam et al. (18) described a similar case in which diaphragmatic herniation developed 2 years after partial resection of diaphragmatic endometriosis without primary closure, underscoring the risk of unreinforced defects Ganesan et al. (19) reported full-thickness diaphragmatic perforations associated with catamenial pneumothorax—contrasting our patient, whose first manifestation was herniation without prior pneumothorax. Notably, pneumothorax occurred only after initial surgical repair, suggesting postoperative remodeling may trigger secondary pleural complications.

Comparable findings were reported by Bobbio et al. (20) who reviewed 20 women surgically treated for diaphragmatic hernia secondary to endometriosis, predominantly affecting the right hemidiaphragm. Their data align with those obtained in the present case, although ours uniquely represents a late diagnosis where thoracic endometriosis was confirmed postoperatively. Similarly, Gaichies et al. (21) described diaphragmatic rupture with hepatic herniation in deep pelvic endometriosis, supporting that chronic infiltration may culminate in structural failure. Unlike their thoracotomy-based repair, our minimally invasive VATS approach reflects the increasing adoption of less invasive surgical strategies; however, as illustrated by the postoperative course in this case, structural repair does not necessarily eliminate the risk of subsequent thoracic complications. Alternatively, previously unrecognized microscopic pleural lesions may have contributed to the development of pneumothorax following surgical intervention.

A similarly extensive thoracic manifestation was reported by Ceccaroni et al. (22) who described a patient with synchronous pericardial, pleural, and diaphragmatic endometriosis associated with severe pelvic and bowel involvement. Their case highlighted the potential for contiguous spread of endometrial tissue through the right hemidiaphragm and adjacent serosal planes, reinforcing the hypothesis of transdiaphragmatic migration via peritoneal fluid dynamics. The authors emphasized that diaphragmatic lesions are often underdiagnosed preoperatively due to their hidden location behind the liver and may coexist with deep pelvic disease, supporting the concept of endometriosis as a systemic, multifocal disorder. Compared to their extensive open approach, our case demonstrates that large diaphragmatic defects and hepatic herniation can be addressed using minimally invasive techniques; nevertheless, long-term disease control may require additional interventions, particularly in the presence of multifocal thoracic endometriosis. To better contextualize the present case within the existing literature, we compiled previously reported cases of diaphragmatic endometriosis–related herniation without prior pneumothorax, detailing initial presentation, hernia size, and pneumothorax history, as presented in Supplementary Table 2.

### 3.3 Pathophysiology and disease spectrum

The current case aligns with the concept of the “extended thoracic endometriosis syndrome” proposed by Larraín et al. (23) who include diaphragmatic hernia among TES variants, even in the absence of pneumothorax or hemothorax. This broader framework increases diagnostic sensitivity and captures atypical presentations such as our patient’s. Large-scale studies also corroborate that diaphragmatic involvement represents an advanced stage of systemic disease. Pagano et al. (11) found diaphragmatic endometriosis in 4.7% of over 1,300 endometriosis patients, mostly with stage III-IV disease and frequent infertility. Similarly, Piriyev and Römer (24) observed diaphragmatic lesions in 1.1% of 8,650 laparoscopic cases, nearly all with deep infiltrating or stage IV endometriosis. In both studies, strong right-sided predominance, severe adhesions and a close association with extensive pelvic involvement were emphasized. These data support the theory of clockwise peritoneal fluid flow and retrograde dissemination of endometrial cells-consistent with our patient’s right-sided presentation. Histopathological confirmation of ectopic endometrial glands and CD10-positive stromal cells within fibrotic diaphragmatic tissue supports chronic hormonally mediated inflammation and progressive tissue weakening, a mechanism incompatible with congenital diaphragmatic defects.

Beyond established theories of retrograde menstruation and peritoneal fluid circulation, increasing evidence highlights the importance of tissue-level and molecular mechanisms in the progression of thoracic endometriosis. In particular, stromal-predominant endometriosis plays a central role in driving chronic inflammation, fibrotic remodeling, and progressive tissue weakening. Endometriotic stromal cells, typically identified by CD10 expression-as observed in the present case-exhibits high inflammatory and angiogenic activity and contribute to extracellular matrix remodeling through cytokine release and fibroblast activation. Recurrent menstrual-cycle–dependent inflammation and microhemorrhage within ectopic endometrial tissue may result in repeated cycles of tissue injury, necrosis, and incomplete repair (3, 25).

Over time, this process can lead to progressive diaphragmatic thinning, loss of tensile strength, and the formation of multiple microfenestrations, consistent with the intraoperative findings in our patient. Unlike acute traumatic rupture, such cumulative damage reflects a chronic, hormonally driven degenerative process. The eventual development of a full-thickness diaphragmatic defect and herniation may therefore represent the end stage of prolonged inflammatory and fibrotic remodeling rather than a sudden mechanical failure. This mechanism aligns with the systemic and progressive nature of advanced endometriosis and helps explain the severity and delayed presentation observed in this case (10, 25).

In addition to the chronic inflammatory and stromal-driven mechanisms previously discussed, optimal medical management-such as long-term use of progestins like dienogest, both alone and in combination with estro-progestins has been shown to improve quality of life and symptom control in patients with endometriosis, which may indirectly influence the natural history of ectopic lesions and their sequelae (26).

### 3.4 Surgical management and multidisciplinary care

The significance multidisciplinary surgical planning is also emphasized in the current study. The protocol established by Nezhat et al. (16) demonstrated that simultaneous video-assisted thoracoscopic surgery (VATS) and laparoscopy allow for comprehensive treatment of combined thoracic and pelvic endometriosis. Our patient underwent staged rather than combined procedures. Although this approach addressed the structural defect, staged management may limit the ability to simultaneously assess and treat multifocal thoracic and pelvic disease. Similar to the conclusions reached by Ceccaroni et al. (27) who analyzed 215 cases over 15 years-most diaphragmatic lesions can be managed laparoscopically, while full-thickness disease-present in only 16% of their cohort-requires thoracic collaboration. The necessity for thoracoscopic rather than laparoscopic repair in our patient verifies this principle.

Ribeiro et al. (28) reported an intraoperative tension pneumothorax during robotic-assisted laparoscopy for diaphragmatic endometriosis, illustrating that diaphragmatic fenestrations may permit gas exchange between the peritoneal and pleural cavities. In our case, the pneumothorax developed after VATS repair, suggesting that postoperative fragility or incomplete sealing may predispose to delayed pleural events. Importantly, surgical repair may have unmasked or facilitated pleural air leakage through residual or newly vulnerable diaphragmatic or pleural areas. Altered intrathoracic pressure dynamics and postoperative tissue remodeling may render previously microscopic or clinically silent pleural or diaphragmatic lesions clinically evident, a phenomenon consistent with the unpredictable behavior of diaphragmatic abnormalities described in thoracic endometriosis (10, 29). These findings highlight the need for careful postoperative monitoring and awareness of thoracic complications following diaphragmatic surgery. The occurrence of postoperative catamenial pneumothorax in this patient underscores that technical repair of a diaphragmatic defect does not necessarily equate to full control of thoracic endometriosis, even when contemporary surgical strategies are applied.

The coexistence of umbilical and diaphragmatic endometriosis in our patient also parallels the case by Yu and Laohathai (30), who proposed retrograde menstruation as a shared pathogenic pathway. While their patient experienced catamenial pneumothorax, ours did not, suggesting that extrapelvic lesions may progress independently. This reinforces the need for thorough inspection of the abdominal wall and diaphragm in patients with either manifestation.

Finally, in recent reviews by Nezhat et al. (3), TES is conceptualized as a systemic, severe form of endometriosis requiring individualized, minimally invasive and multidisciplinary care. Conservative management remains first-line, with surgery reserved for refractory or progressive cases. In our case, delayed diagnosis and the lack of early coordinated care likely allowed progression to full-thickness rupture. Postoperative hormonal therapy, consistent with current recommendations, was implemented; however, as demonstrated by the recurrence of pneumothorax, medical suppression alone may not fully prevent thoracic reactivation in advanced disease.

Surgical management of thoracic and diaphragmatic endometriosis remains heterogeneous, and no standardized recommendations exist regarding the optimal extent of diaphragmatic repair or pleural intervention. Reported surgical approaches range from primary suture repair to mesh reinforcement, depending on defect size, tissue quality, hormonal activity, and intraoperative findings. Importantly, the routine use of prosthetic materials for diaphragmatic repair in hormonally active endometriosis remains controversial due to concerns regarding foreign-body reaction, adhesion formation, and potential difficulties during future reoperations (22).

Similarly, the role of prophylactic pneumothorax-preventive procedures, such as extensive pleurectomy or complete pleurodesis, is not clearly defined in patients without a preoperative history of pneumothorax. Existing literature suggests that these interventions are most commonly reserved for patients with recurrent or established catamenial pneumothorax, while their routine use during initial surgical management of diaphragmatic endometriosis remains debated. As a result, surgical strategies are typically individualized and guided by clinical presentation, disease extent, and multidisciplinary assessment rather than uniform protocols (10, 20, 27, 29).

### 3.5 Implications for practice

This case and the reviewed literature converge on several critical implications. First of all, diaphragmatic endometriosis is rarely isolated; it is typically part of extensive, high-stage pelvic disease. Routine inspection of both hemidiaphragms during laparoscopy should become standard in patients with advanced or atypical symptoms. Secondly, delayed or incomplete repair can lead to catastrophic outcomes such as herniation, underscoring the need for early multidisciplinary management involving both gynecologic and thoracic surgeons. Third, postoperative follow-up and hormonal therapy are essential components of long-term management and may reduce recurrence risk.

In the context of comprehensive patient care, both surgical and medical therapies are integral to long-term management of endometriosis. For example, prospective studies have shown that both progestin monotherapy and progestin/estrogen combination regimens (e.g., dienogest alone or combined with ethinylestradiol) can significantly reduce pain and improve quality of life in women with endometriosis, highlighting the importance of individualized, multimodal strategies in managing this chronic condition (31). Moreover, coordinated care pathways should be established to ensure continuity between surgical, gynecologic, radiologic and psychological teams. Shared decision-making, standardized imaging protocols and structured long-term follow-up may help bridge current gaps in care and reduce diagnostic delays. Finally, awareness of atypical TES manifestations-such as chronic right upper quadrant pain or referred shoulder pain-can facilitate earlier recognition and improve patient outcomes through timely, team-based interventions. To enhance generalisability, a schematic illustration of a proposed pelvic–thoracic endometriosis multidisciplinary care pathway is provided in the Supplementary Figure 1.

## 4 Conclusion

This case underscores the diagnostic and therapeutic challenges of diaphragmatic endometriosis, particularly when complicated by herniation. Early multidisciplinary recognition and intervention are essential to limit progression and reduce the risk of severe complications. However, as illustrated in this patient, structural surgical repair does not necessarily equate to complete control of thoracic endometriosis, and postoperative recurrence may occur despite adherence to contemporary management strategies. Our findings also emphasize the necessity of comprehensive, patient-centered follow-up, including hormonal management and long-term surveillance. Our findings highlight that while thoracic endometriosis represents the broader clinical spectrum of extrapelvic disease, diaphragmatic endometriosis, in particular, should be recognized as a sentinel manifestation of advanced pelvic pathology-one that demands systematic inspection, early multidisciplinary management and individualized surgical planning to prevent progression to full-thickness rupture or herniation.

Beyond surgical and medical care, diaphragmatic and thoracic endometriosis significantly affect patients’ quality of life, often causing chronic pain, fatigue and psychological distress. Integrating psychological support and patient education into routine management may improve coping, treatment adherence and overall wellbeing. Increased awareness and refined diagnostic pathways may facilitate earlier recognition and support more effective long-term disease control.

## Data Availability

Data are not publicly available due to privacy and ethical restrictions, but all relevant information is provided within the article.
