# Supplementary material for: Diaphragmatic endometriosis with hepatic herniation: case report and review of literature
Source: Front Med (Lausanne). 2026 Apr 1;13:1740350. doi: 10.3389/fmed.2026.1740350 (PMC13079330; doi:10.3389/fmed.2026.1740350)
Supplement: Supplementary file 1 [file Data_sheet_1.docx]

## Supplementary Table 1. List of medications used in treatment

## The table summarizes all hormonal therapies and intrauterine devices (IUDs) used by the patient throughout the clinical course, including therapeutic effects, tolerability, and treatment transitions.

| **Period (years)** | **Medication / Device** | **Type** | **Dose / Route** | **Duration** | **Clinical effect** | **Remarks** | **Reason for change or discontinuation** |
| --- | --- | --- | --- | --- | --- | --- | --- |
| ~1998–2001 | Combined oral contraceptives (unspecified) | Estrogen–progestin oral contraceptive | PO, 1 tab daily | ~3 years | Menstrual suppression, pain reduction | Well tolerated | Discontinued due to pregnancy planning |
| 2001–2006 | — | — | — | 5-year break (pregnancies) | — | — | — |
| 2006–2008 | Cerazette | Desogestrel 75 µg, progestin-only pill | PO, 1 tab daily | ~2 years | Reduced menstrual pain | Occasional spotting | Irregular bleeding |
| 2008–2009 | Evra® patch | Combined transdermal contraceptive (EE/norelgestromin) | Transdermal, 1 patch/week | ~1 year | Stable hormonal control | Mild local irritation | Switch to IUD |
| 2009–2014 | Mirena® LNG-IUS (first) | Levonorgestrel-releasing IUD 52 mg | Intrauterine | 5 years | Substantial reduction of bleeding and dysmenorrhea | Minimal | Routine removal after 5 years |
| 2014–2016 | — | — | — | ~2 years | Post-removal, miscarriage episode reported | — | — |
| 2016–2021 | Mirena® LNG-IUS (second) | Levonorgestrel-releasing IUD 52 mg | Intrauterine | ~5 years | Improved cycle control and pain | Mild initial spotting | Elective removal end of 2021 |
| Early 2022–present | Mirena® LNG-IUS (third) | Levonorgestrel-releasing IUD 52 mg | Intrauterine | Since 2022 | Maintained pain control | — | Currently in situ |
| Jan 2023 – Sep 2025 | Diemono® | Combined oral contraceptive (EE/drospirenone) | PO, 1 tab daily | ~2.5 years | Effective hormonal suppression; used concomitantly with IUD before surgery (Sep 2023) | Slight mood changes | The patient experienced ovulation despite having only one ovary and taking Diemono, as well as reported chest pain.  Switched to GnRH antagonist |
| Since Sep 2025 | Ryeqo® | Relugolix 40 mg + Estradiol 1 mg + Norethindrone 0.5 mg | PO, 1 tab daily | Ongoing | Good pain control and tolerance | — | Current therapy |

Abbreviations: EE – ethinylestradiol; IUD – intrauterine device; LNG-IUS – levonorgestrel intrauterine system; GnRH – gonadotropin-releasing hormone.

**Supplementary table 2.** Comparative summary of reported cases of endometriosis-related diaphragmatic herniation

| **Author (Year)** | **Study Type** | **Initial Presentation** | **Hernia Size** | **Hepatic Herniation** | **Prior/Concomitant Catamenial Pneumothorax** |
| --- | --- | --- | --- | --- | --- |
| Bobbio et al. (2024) | Case series (n=20) | Cyclical thoracic pain (7/20) or pneumothorax (13/20) | Median 8 cm (range 2.5–22 cm) | Yes (in 18/20 right-sided cases) | Absent in a subset (5/20 cases presented with isolated herniation without prior pneumothorax) |
| Gaichies et al. (2019) | Case report | Cyclical scapular and pelvic pain | 4.5 cm defect | Yes | No (no pneumothorax reported) |
| Ceccaroni et al. (2012) | Case report | Severe right upper-quadrant and shoulder pain | No hernia; extensive full-thickness diaphragmatic infiltration | No (no visceral displacement) | No (dominant symptoms were pain and pelvic disease) |
| Ganesan et al. (2023) | Case report | Shortness of breath and hemoptysis | Multiple full-thickness fenestrations | No (fenestrations without visceral herniation) | Yes (recurrent catamenial pneumothorax) |
| Islam et al. (2024) | Case report | Cyclic and later noncyclic right upper quadrant pain | <5 cm defect | Yes | No (herniation developed 2 years after prior surgery, no pneumothorax reported) |
| Present case | Case report | Chronic cyclical pain for >20 years (misdiagnosed as musculoskeletal) | Large 6 x 8 cm defect | Yes (partial herniation of segments VII and VIII) | No (first pneumothorax episode occurred only after surgical repair) |


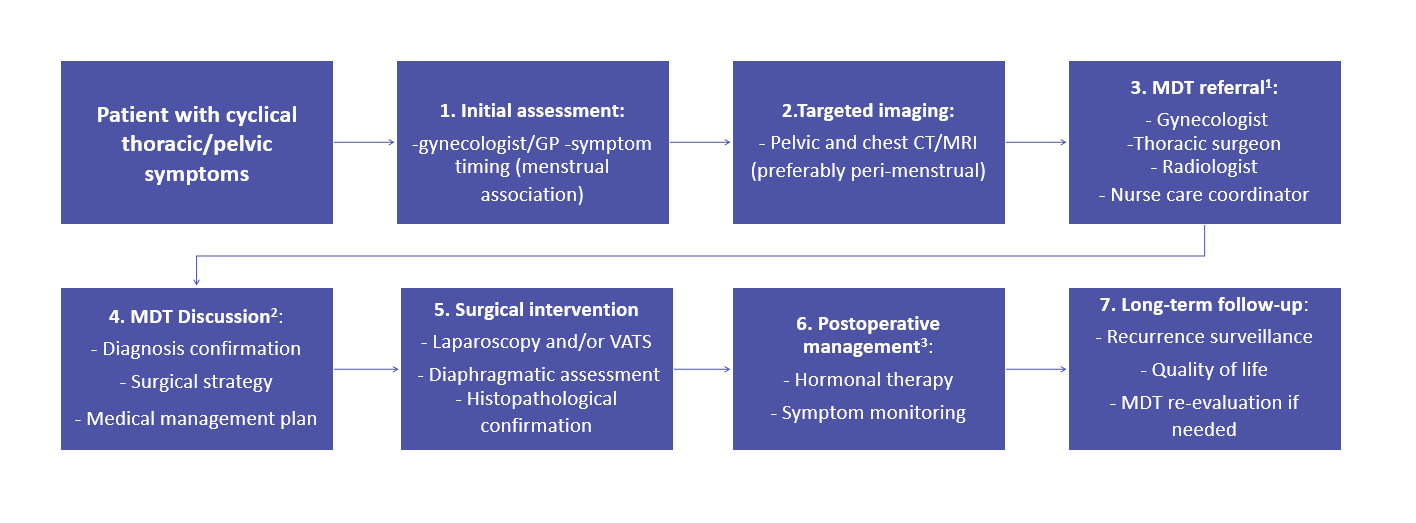


Supplementary figure 1. Proposed multidisciplinary pelvic–thoracic endometriosis care pathway

*Note:*

^1^Early referral recommended

^2^Timely MDT discussion

^3^Prompt initiation of postoperative hormonal therapy
